# Mental Wellbeing Impact Assessment (MWIA) in the workplace

**DOI:** 10.1108/JPMH-01-2017-0002

**Published:** 2017-09-18

**Authors:** Charlotte Burford, Silvia Davey, Alec Knight, Sadie King, Anthea Cooke, Tony Coggins

**Affiliations:** 1 Maudsley International, Institute of Psychiatry, King’s College London, London, UK; 2 King’s Improvement Science, Institute of Psychiatry, King’s College London, London, UK; 3 The Tavistock Institute, London, UK; 4 SLaM and Maudsley International; 5 South London and Maudsley NHS Foundation Trust, London, UK

**Keywords:** Wellbeing, Impact assessment, Workplace, MWIA

## Abstract

**Purpose:**

The Mental Wellbeing Impact Assessment (MWIA) is an evidence-based tool that guides decision makers, such as policy makers and service managers, about the potential impacts of a new programme or policy change. It was initially used in urban regeneration but has subsequently been used in housing, children’s centres and education. The purpose of this paper is to report, for the first time, on the strengths and weaknesses of using the MWIA in the workplace.

**Design/methodology/approach:**

Feedback was collected from staff who participated in stakeholder workshops as part of the MWIA process at two different public sector organisations.

**Findings:**

The MWIA can be used as an effective workplace assessment tool and is valuable as both a diagnostic tool and as an intervention in its own right. The MWIA generates tailored action plans focussed on addressing the organisation or team-specific issues. The weaknesses of the MWIA in the workplace are mainly focussed around management cooperation and commitment to the process which should be screened for prior to engaging in the full stakeholder workshop.

**Originality/value:**

This is the first report of MWIA’s use in the workplace but suggests that it is a useful tool which can be used to support workplace wellbeing, especially in relation to a policy or organisational change. Further studies should be carried out to fully understand the impact of the MWIA in the workplace.

## Introduction

Mental Wellbeing Impact Assessment (MWIA) was developed over a ten-year period by the South London and Maudsley NHS Foundation Trust and partners (MWIA National Collaborative). It has been used both nationally and internationally (West and Scott-Samuel, 2010). MWIA is an evidence-based diagnostic tool that aims to orientate decision makers such as policy makers and service managers towards considering the impact of policies, programmes and services on the mental wellbeing of the target group. MWIA is a stepwise process that begins with a desk-based screening tool and culminates in a workshop that engages multiple stakeholders and results in co-produced action plans. The action plans aim to develop the positive impacts on wellbeing and mitigate against any negative impacts.

MWIA was developed based on the health impact assessment methodology (European Centre for Health Policy, 1999), an evidence review about mental wellbeing influences that includes a social model of health and takes an assets-based approach. Initially, it was used in urban regeneration but has since been used in housing, children’s centres and education. In addition, over the past three years, we have been exploring, and finding success, in its application in workplace wellbeing.

The economic benefits associated with improved workplace wellbeing, in addition to the benefits to the individual, make it an increasingly attractive area to employers. Employees who are physically and psychologically healthy are more productive, better at decision making, have reduced absenteeism and reduced healthcare costs, have increased resilience, engage more and are better at coping with uncertainty and change (Boorman, 1999; Hillier *et al.*, 2005; National Institute of Clinical Excellence (NICE), 2009).

Employers have previously approached wellbeing in a reactive rather than proactive manner. It has however been shown that interventions focussed on worker wellbeing can have a significant effect on commercial outcomes, and an effective human capital management is starting to be seen as indicative of companies’ long-term prospects (Litchfield *et al.*, 2016). For example, it has been estimated that every pound spent on prevention and early intervention can result in ten pounds of savings for businesses, which are currently estimated to spend £554 per person on employee absences (Chartered Institute of Personnel and Development, 2015).

Major factors that directly affect the employee’s wellbeing and productivity include an organisational change and stress, in addition to relationships with employers, working arrangements, relationships at work and an employee’s work design and work demands (Hillier *et al.*, 2005; Campaign for Social Science, 2013; Litchfield *et al.*, 2016). Current workplace mental wellbeing interventions therefore range from approaches that focus on the individual’s ability to cope, endure stress and proactively manage a healthy work-life balance (resilience) to organisational and wider system-level approaches that consider structural and cultural aspects of organisational culture such as the access to work-based support, engagement and mental health awareness (Gravelling *et al.*, 2008; Public Health England, 2014).

Stress management interventions, which focus on stress awareness and the development of techniques to minimise the impact of stressors, form a large part of workplace wellbeing programmes (Seymour and Grove, 2005; Public Health England, 2014). In addition, manager training has been shown to benefit the mental wellbeing of those who they oversee (Tsutsumi, 2011) and Mental Health First Aid training for managers has also been shown to be effective in improving the mental wellbeing of those who partake in it (Kitchener and Jorm, 2004). At the structural level, interventions such as flexible working hours, work-based support schemes, line manager training in mental wellbeing and increased staff participation can improve the workplace wellbeing (Gravelling *et al.*, 2008).

MWIA is a unique approach that operates across the continuum of the above described interventions. It uses a reflective practice to identify underlying issues and the interventions that would be most appropriate for each specific set of circumstances. In this respect, it is both a diagnostic tool and an intervention in its own right.

**Figure 1:**
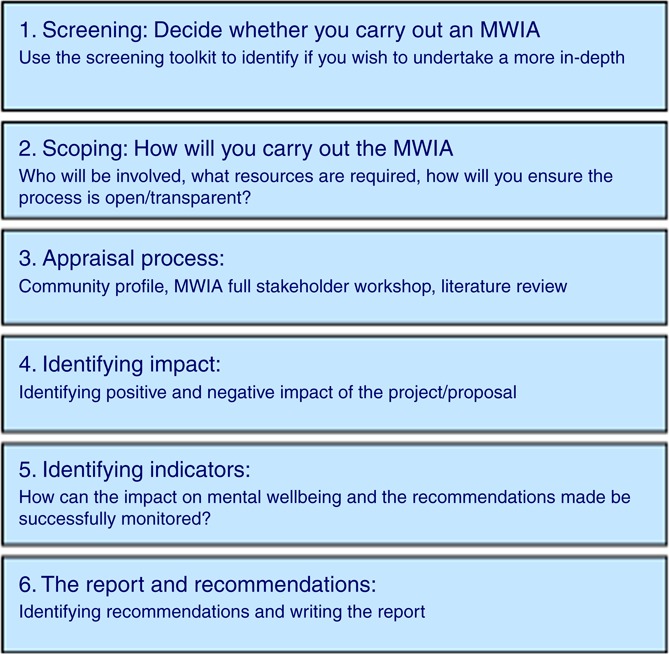
Stages of the MWIA process as applied in the workplace

An MWIA begins with a “screening” phase (Figure 1). In the screening phase, the proposal or issue is explored with a small number of people who represent different viewpoints such as frontline staff as well as management. Typically, the manager or human resources lead responsible for the proposal is present for the screening. This initial discussion provides the context and begins to unpack the potential impact on the wellbeing of a proposal or issue, and can lead to an action plan in response to any potential adverse impacts identified. The screening considers the same issues that will be discussed more completely as part of the full stakeholder workshop and can be found in the MWIA toolkit (Cooke *et al.*, 2011).

The “screening” informs the “scoping” phase, which determines whether further investigation in the form of a full MWIA is necessary, given the possible impacts identified. In addition, the scoping identifies: the resources and time required to undertake the project; if those participating would have a safe space to have this engagement; and whether the findings from the MWIA would be taken on board by the organisations concerned. This requires in-depth conversations with the management of the workplaces in question and an honest appraisal of their commitment to supporting the outcomes of a full stakeholder workshop.

If a full MWIA is indicated, then a stakeholder workshop will be conducted in which the toolkit is used (Figure 1) to stimulate a discussion amongst those who are being affected by a proposal or issue. The toolkit asks the participants to consider how different factors, which are known to be associated with mental wellbeing, are being or will be impacted on. The toolkit asks the participants to consider both positive and negative impacts on these factors (Knapp *et al.*, 2011). Actions to minimise the negative impacts and maximise the positive are then discussed and indicators are developed to assess the outcome of these actions. A short literature review is conducted after the workshop to analyse the suggestions in the context of best practice as well as examining demographic data (community profiling) of the affected population.

## Methods

Two case studies are presented to help illustrate areas where the MWIA has worked well in workplaces and how it could be improved. These organisations underwent an MWIA between 2013 and 2014.

The first organisation was an academic health sciences collaboration centre composed of member organisations from different backgrounds such as clinical services (including NHS trusts), academic research and human resources. The organisation underwent the MWIA process as a result of funding awarded to improve the workplace mental wellbeing of staff. The seven teams that underwent the initial screening were from a range of different services and therefore faced a diverse range of issues. The experiences of staff undergoing the MWIA process was collected from the final report produced after the workshop and from the interactions with facilitators (TC, AC, SK).

The second organisation was a local authority. Similar to case study one, the local authority was composed of teams operating within different areas of the organisation and also included human resources personnel. A total of seven teams were also included in an initial screening process and six went on to complete a full MWIA workshop. During the screening phase, it was recognised that the environment within the seventh team would not enable an effective full stakeholder workshop and generate workable action plans due to issues around the relationships between the team members and management. The experience of the participants who underwent the workshops was collected by a telephone interview one year after the workshop was held (see the Appendix).

Interviewers had experience in facilitating a full MWIA stakeholder workshop and had been trained to deliver the MWIA screening (CB) or were involved in the original development of the MWIA (AC). Both interviewers were contracted to work on MWIAs by Maudsley International (MI) at the time of interviewing. In total, four participants were contacted by e-mail from different teams within the local authority to share their experiences anonymously. All four participants gave a full telephone interview and were aware that the interviewers worked for MI. The four team members were selected as they took part in both the initial screening and the full stakeholder workshops. The participants and researchers were both alone when the telephone interviews were being conducted. Written notes were taken during the interviews which lasted between 30 and 45 minutes.

The responses from participants in both case studies were compared and issues that were common to both were identified.

## Results

The evidence from the case studies suggests that the MWIA is a useful tool for teams to assess and discuss the issues around wellbeing. It also enables the joined development of solutions that may require organisational-level change, and therefore commitment from the higher levels of management. MWIA’s external facilitation aids coordination, but also provides a safe space for the participants to freely voice their concerns that may affect their workplace wellbeing.

### The MWIA screening toolkit can be used as a standalone assessment

As the screening involves a small group of people, it will not capture the full range of issues impacting, or potentially impacting, on teams. However, it may start to guide managers towards thinking about these challenges and it can often generate enough information to inform the development of actions in response to the findings it generates. For example, one of the teams within the health sciences centre felt that, based on the issues raised in the screening, there were issues related to the team’s environment that made it difficult to provide a safe space for the employees to discuss issues as part of a full stakeholder workshop. The screening acted as a stimulus to bring about a change in the management of this team and was therefore useful in its own right.

Additionally, employees from the local authority underwent training in delivery of the screening toolkit, following the full stakeholder workshop, so that it could be delivered to groups of employees from large teams (bigger than ten) who could not all be included in a full workshop, due to resource constraints. Unlike the full MWIA that tends to be delivered to groups of ten or smaller, the MWIA screening toolkit has the potential to be used in large teams (Cooke and Stansfield, 2009). This is important, given that the smaller number of individuals included in a full stakeholder workshop may not represent the diverse views held in larger teams.

In both case studies, it was noted that the screening alone increased the understanding and awareness of mental wellbeing, even if the participants did not undergo the full MWIA process.

### MWIA requires the management to be committed to and engaged with the process

One of the teams in the local authority that underwent a full stakeholder workshop was restructured shortly after the process was completed. A year later, the actions proposed following the MWIA process had not been implemented due to uncertainty and continued disruption to the team. The upcoming restructuring should have been identified as a barrier to completing the full stakeholder workshop.

Engagement with senior management during the implementation of the actions identified by the MWIA was highlighted as important for successful outcomes in both case studies. One of the teams in the local authority highlighted the benefit of receiving management feedback on suggestions – even when nothing could be done about the issue. The explanation helped increase employees’ sense of control and improved wellbeing. This team went on to introduce a regular line manager newsletter, which reported the outcomes of anonymous employee suggestions in the previous month. Furthermore, in the health sciences centre, the repeated clarification of progress with implementation of the actions identified in the MWIA demonstrated that it was not another “tick-box” exercise, as some employees had initially assumed, but was able to generate a real change. In the local authority, some employees were initially sceptical of the process and the likely benefit, especially given the time commitment it required from the employees. Previously, similar work had been started but never completed within the organisation.

In addition, management support can be important if issues related to organisational culture are raised by the MWIA stakeholder workshop. Within the NHS Trust team, it was felt that their current working conditions were inconsistent with the values of the Trust and the values the individuals themselves held, which had motivated them to train in their respective careers. At the team level, the action suggested was for the line manager to acknowledge that the team were facing difficulties and to give more praise to the team members for the good work they were doing in difficult working conditions. At the organisational level, cultural change would be needed to address the inconsistencies between daily practices and trust values, which requires support from the highest levels of management.

### MWIA generates solutions that give responsibility back to teams

Although the MWIA can generate issues requiring an organisational-level change, many of the actions identified place emphasis on the team and what the team and individuals can do to improve their own wellbeing. For example, in one of the teams within the local authority, it was suggested that staff were not being recognised for their good work. The team therefore decided to set up a display on the wall, and every time they received some good feedback, it was added to the wall to help improve the team morale. This action helped to motivate the members of the team and encouraged them to be more engaged, although it was not costly and did not require a management input. In the health sciences centre, it was felt strongly that there was an unhealthy work-life balance and this was impacting negatively on the home-life of members of staff. It was suggested that one of the ways of helping staff cope would be to establish peer-to-peer relationships within teams that could provide valuable support.

### MWIA provides external facilitation

In both case studies, external facilitators delivered the MWIA, which was regarded as very important to the success of the process. It allowed a group-led approach with the stakeholders providing “the content and direction” and the facilitators “just adding structure”. Ensuring managers were not present allowed an open forum for discussion, which meant that the issues could be fully explored in a safe environment. This was important in one of the teams in the health sciences centre, due to sensitivities between team members and management. Conversely, in the local authority, the manager who commissioned the work was present at the full stakeholder workshop. The manager was moved between different teams during the day and it was highlighted that this was important for allowing employees to speak freely. However, the manager also felt that it was useful to be able to listen to those she managed and the issues they faced. The participants felt that the external facilitation provided an opportunity to speak honestly and openly in a safe environment. It was mentioned that during times of organisational change or difficulty, individuals can feel reluctant to express fears and share those with others, especially if there are concerns about job security. This was the case for one team in the local authority and the external facilitation was felt to be particularly important.

### MWIA may initially raise issues that lead to more stress

The nature of the MWIA process means it can often bring up some very personal issues and this may initially cause a higher strain on mental wellbeing before they resolve. For example, in the local authority, there was an individual who decided, shortly after the MWIA full stakeholder workshop, that they would leave the organisation. It was felt that the MWIA process helped the individual reach that decision as it became clear that the issues most important to them were outside the control of the organisation. It was felt that MWIA had helped to make it a positive decision for the individual, and the manager felt that it was a positive decision as well.

### MWIA as an intervention in its own right

Participants felt that the MWIA workshop gave them the opportunity to have their voice heard by senior management and that they were allowed to express concerns. In addition, it helped team members appreciate issues that others within the team were facing, such as age, circumstances in their personal life and career aspirations and ambitions. The sharing of perspectives helped team members to understand what motivates others and was beneficial for contextualising the behaviour and interactions between team members. In both the NHS Trust and local authority, stakeholders felt that it was beneficial to learn that others felt the same way and shared similar concerns.

### MWIA as a diagnostic tool

In both case studies, the actions suggested by the groups differed depending on the specific challenges the organisations were facing. For example, teams within both organisations had issues with the physical space of the workplace; in the local authority, it related to a recent move to a new space, whilst in the NHS Trust, it related to the physical layout. The actions the teams identified were therefore tailored to the specific problems rather than just applying a blanket solution to issues.

## Discussion

The findings from the case studies support the use of the MWIA as both an intervention in its own right and a diagnostic tool. The process of sharing and exploring concerns can help improve mental wellbeing as it allows an individual to define the issues really impacting them (The Mental Health Foundation, 2016). The MWIA stakeholder workshop provides a forum for team members to do this and can therefore be of benefit even in teams unable to implement the recommended actions. The other role of the MWIA, as a diagnostic tool, is perhaps one of its most useful aspects. It helps to identify actions in response to the discussed issues, which are likely to make the greatest difference as they are generated based on the employee experience rather than being instituted in a top-down manner. Both organisations could have rolled out stress management training to employees, although this would not have addressed the most salient issues. There is no “one-size fits all” for mental wellbeing and it depends on the organisational culture and values. The MWIA can be used to “diagnose” the real problems affecting staff so the organisations’ time and efforts to improve mental wellbeing can be used more effectively.

In addition, the process can help to highlight the issues that may be impacting different members of the team and can raise the awareness of mental wellbeing in general. Increasing the awareness of good mental wellbeing can help lead to an early detection of mental health problems, which, in turn, leads to earlier intervention and better outcomes (NICE, 2009).

A challenge with the MWIA process is ensuring that the actions are implemented. When an organisation or team is unable to implement the actions suggested, it can cause individuals to feel more helpless, as they feel ignored. This can lead to a decreased sense of control and poorer mental wellbeing at work (Public Health England, 2014). Therefore, the MWIA should only be used in circumstances where there is an organisational support; the scoping phase can also be used for developing an understanding of whether an organisation is in a position to support a full MWIA workshop. In the case of the local authority, a more in-depth scoping phase would hopefully have identified the upcoming restructuring that had been planned for one of the teams, and therefore highlighted why this may have made them unsuitable for a full stakeholder workshop.

Paradoxically, MWIA also provides an opportunity to increase employees’ sense of control and wellbeing through the implementation of actions. Frequently, the actions proposed can be carried out by the employees without the need for a manager input. This can help provide a sense of control and empowerment as the individual, or individuals, become responsible for driving the change.

The case studies also demonstrate the challenging and complex nature of some of the issues raised by MWIA. It is important to explore this with management and make them fully aware of this possible outcome during the screening and scoping phase. This allows them to prepare for how they might navigate any potential issues.

Finally, it is important that quantitative methods for assessing the impact of MWIA are developed. During the MWIA workshop, participants are encouraged to decide on indicators for each of their proposed actions that will act as the measures of their success. Possible measures suggested in the local authority included number of sick days taken, staff retention and promotion, and number of staff suggestions submitted. However, it is important that quantitative measure can be applied across different organisations to allow the comparison of the impact of MWIA. The local authority suggested using the Warwick-Edinburgh Mental Wellbeing Assessment Scale (Tennant *et al.*, 2007) to assess mental wellbeing before and after the workshop. A difficulty with this is that mental wellbeing may still decrease after the workshop, as a result of the change that necessitated the workshop in the first place, and it is not possible to know what mental wellbeing would have been like without the MWIA.

The experiences in these two cases studies suggest that the MWIA can be used as an effective assessment tool for workplace wellbeing. In both case studies, the MWIA toolkit was applied in full but highlighted how the screening alone can add value. In addition, the MWIA can be used to identify issues most important to the employees and therefore allow tailored solutions, which could save the organisations’ time and money over providing generic, “off-the shelf” mental wellbeing interventions.

The weaknesses of the MWIA are mainly related to the implementation of actions and depend on management cooperation. It may therefore be necessary to develop the engagement process so that organisations have a thorough understanding of what they are committing to. Finally, the assessment of the impact of the MWIA needs to be developed further. Although this is considered within the process, with participants trying to develop indicators for each of the actions they propose, it would be useful to be able to assess any overall global changes in mental wellbeing as a result of undertaking the MWIA.

It is important to note that results derived from case studies cannot be generalised, and are specific to the one particular case. In addition, bias and, in particular, observer bias and the “halo” effect may have impacted the results. More work is therefore needed to develop robust evaluation methodologies for the impact of the MWIA. The use of the MWIA in the workplace is in its infancy but these case studies suggest that it has the potential to provide an effective means of improving a workplace mental wellbeing (King, 2014).

## Appendix

1. Has an action plan been completed?
2. Have any of the actions been implemented successfully? If so, how many and which ones?
3. Do you foresee any difficulties in implementing any of the actions? If so, please describe.
4. What difference did the MWIA make in understanding the wellbeing issues of your team/issue?
5. How would you have tackled the issue your service/team was experiencing without MWIA?
6. Do you think you would have found the same answers without MWIA? Why do you think this is?
7. Do you think you may use MWIA in the future and why?
8. What other impact assessment or team/service planning tools do you use? Do you think MWIA can be used with these/instead of these/be integrated?
9. Did MWIA provide a way of clarifying and being transparent around decisions? Can you give an example.
10. What worked really well?
11. What did you like least/most about the process?
12. Were there any objections to the process before or after?
13. Did the MWIA raise any difficult issues?
14. Did you/participants feel is was a safe space to talk openly?
15. How were the difficult issues dealt with during and after?
16. Were there any unexpected consequences.
17. Were you able to get appropriate support for all the issues that needed to be addressed?
18. Did you feel you/your team/your organisation has enough recourses (money/time/skills and experience) to implement the required changes?
19. Was there any stigma attached to the use of MWIA?
20. Have there been further opportunities after the MWIA to talk more/work more on mental wellbeing?
21. Any strategic changes connected to working with mental wellbeing? For example, policy, new boards or staff forums, new partnerships?
